# Hydrogen bonding in duplex DNA probed by DNP enhanced solid-state NMR N-H bond length measurements

**DOI:** 10.3389/fmolb.2023.1286172

**Published:** 2023-12-04

**Authors:** Lakshmi Bhai, Justin K. Thomas, Daniel W. Conroy, Yu Xu, Hashim M. Al-Hashimi, Christopher P. Jaroniec

**Affiliations:** ^1^ Department of Chemistry and Biochemistry, The Ohio State University, Columbus, OH, United States; ^2^ Department of Chemistry, Duke University, Durham, NC, United States; ^3^ Department of Biochemistry and Molecular Biophysics, Columbia University, New York, NY, United States

**Keywords:** hydrogen bonding, DNA, Watson-Crick and Hoogsteen base pairs, dynamic nuclear polarization, magic angle spinning solid-state NMR

## Abstract

Numerous biological processes and mechanisms depend on details of base pairing and hydrogen bonding in DNA. Hydrogen bonds are challenging to quantify by X-ray crystallography and cryo-EM due to difficulty of visualizing hydrogen atom locations but can be probed with site specificity by NMR spectroscopy in solution and the solid state with the latter particularly suited to large, slowly tumbling DNA complexes. Recently, we showed that low-temperature dynamic nuclear polarization (DNP) enhanced solid-state NMR is a valuable tool for distinguishing Hoogsteen base pairs (bps) from canonical Watson-Crick bps in various DNA systems under native-like conditions. Here, using a model 12-mer DNA duplex containing two central adenine-thymine (A-T) bps in either Watson-Crick or Hoogsteen confirmation, we demonstrate DNP solid-state NMR measurements of thymine N3-H3 bond lengths, which are sensitive to details of N-H···N hydrogen bonding and permit hydrogen bonds for the two bp conformers to be systematically compared within the same DNA sequence context. For this DNA duplex, effectively identical TN3-H3 bond lengths of 1.055 ± 0.011 Å and 1.060 ± 0.011 Å were found for Watson-Crick A-T and Hoogsteen A (syn)-T base pairs, respectively, relative to a reference amide bond length of 1.015 ± 0.010 Å determined for N-acetyl-valine under comparable experimental conditions. Considering that prior quantum chemical calculations which account for zero-point motions predict a somewhat longer effective peptide N-H bond length of 1.041 Å, in agreement with solution and solid-state NMR studies of peptides and proteins at ambient temperature, to facilitate direct comparisons with these earlier studies TN3-H3 bond lengths for the DNA samples can be readily scaled appropriately to yield 1.083 Å and 1.087 Å for Watson-Crick A-T and Hoogsteen A (syn)-T bps, respectively, relative to the 1.041 Å reference peptide N-H bond length. Remarkably, in the context of the model DNA duplex, these results indicate that there are no significant differences in N-H···N A-T hydrogen bonds between Watson-Crick and Hoogsteen bp conformers. More generally, high precision measurements of N-H bond lengths by low-temperature DNP solid-state NMR based methods are expected to facilitate detailed comparative analysis of hydrogen bonding for a range of DNA complexes and base pairing environments.

## Introduction

Key biological processes and mechanisms involving DNA, including transcription and replication, depend on the details of hydrogen bonding within guanine-cytosine (G-C) and adenine-thymine (A-T) DNA base pairs (bps) (Goodman, 1997). DNA bps typically adopt canonical Watson-Crick (WC) conformations, but can also take on alternate, thermodynamically less stable (by ∼3 kcal/mol) Hoogsteen (HG) hydrogen bonding topologies (Nikolova et al., 2011), obtained by rotating the purine base by 180° around the glycosidic bond. The latter have been shown to exist transiently in naked double-stranded DNA with lifetimes on the order of ∼0.1–1 ms and populations on the order of ∼0.1–1% (Nikolova et al., 2011) and, remarkably, can feature as major bp conformers at specific sites in complexes of B-DNA with proteins and drug molecules and in DNA sequences containing damaged nucleotide bases where they assume key roles in DNA recognition, damage accommodation, repair and replication (Patikoglou et al., 1999; Ling et al., 2003; Nair et al., 2004; Cuesta-Seijo and Sheldrick, 2005; Kitayner et al., 2010; Lu et al., 2010; Sathyamoorthy et al., 2017; Shi et al., 2018).

Comprehensive characterization of base pairing and hydrogen bonding in DNA systems in their native environment presents considerable difficulties for many experimental techniques. X-ray crystallography and cryoelectron microscopy typically cannot directly visualize the locations of hydrogen atoms, precluding detailed determination of hydrogen bond geometries, and can also suffer from insufficient electron density at the DNA sites leading to ambiguity in distinguishing WC and HG bp conformers (Wang, 2005; Kitayner et al., 2010; Hintze et al., 2017; Shi et al., 2021); additionally, for certain systems the former may be further complicated by potential alterations in bp conformation related to crystal packing (Abrescia et al., 2004). While solution-state NMR spectroscopy is an exquisite tool for probing DNA bp conformations based on characteristic chemical shifts (Nikolova et al., 2011; Shi et al., 2018; Xu et al., 2018) and purine-pyrimidine N-H···N and N-H···O hydrogen bonds via measurements of internucleotide ^2h^J_NN_, ^1h^J_NH_ and ^3h^J_NC_’ coupling constants (Dingley and Grzesiek, 1998; Pervushin et al., 1998; Dingley et al., 1999; 2000; Majumdar et al., 1999; Kojima et al., 2000; Barfield et al., 2001; Majumdar and Patel, 2002; Grzesiek et al., 2004), intranucleotide ^1^J_NH_ coupling constants (Dingley et al., 1999; Barfield et al., 2001; Ishikawa et al., 2001; Manalo et al., 2005), internucleotide ^1h^D_NH_ residual dipolar couplings (Wu et al., 2001), and cross-correlated (Chiarparin et al., 2001; Riek, 2001) and longitudinal (Bytchenkoff and Bodenhausen, 2003) relaxation rates, application of this methodology to large, slowly tumbling DNA-protein complexes and assemblies presents significant challenges. On the other hand, details of base pairing and hydrogen bonding in such large DNA systems can in principle be probed by complementary techniques including infrared (IR) spectroscopy (Stelling et al., 2019; Fick et al., 2021; Peng et al., 2023) and solid-state NMR (DiVerdi and Opella, 1982; Leppert et al., 2004; Riedel et al., 2005; Sergeyev et al., 2011; Daube et al., 2019; Conroy et al., 2022), which generally are not hampered by limitations related to molecular size and, as necessary, can be combined with isotope labeling of select residues for site-specific resolution.

Previous studies by others and us (Sergeyev et al., 2011; Wenk et al., 2015; Wiegand et al., 2017; Jaudzems et al., 2018; Daube et al., 2019; Conroy et al., 2022; Elathram et al., 2022) have demonstrated that solid-state NMR enhanced by low-temperature dynamic nuclear polarization (DNP) (Barnes et al., 2008; Jaudzems et al., 2019) is a particularly useful tool for atomic level characterization of nucleic acids and their complexes with small molecules and proteins under native-like conditions. In this context, we have recently shown that WC and HG bps can be distinguished from one another based on characteristic ^13^C and ^15^N chemical shifts and internuclear dipolar couplings determined in DNP solid-state NMR spectra for DNA-protein complexes as large as the nucleosome core particle (Conroy et al., 2022). Here we explore the possibility of gaining additional insights into the details of hydrogen bonding in DNA systems by using DNP solid-state NMR based methods.

In principle, N-H···N hydrogen bonds for A-T and G-C bps in ^15^N-enriched DNA systems can be probed via solid-state NMR measurements of N-N correlations in multidimensional chemical shift correlation spectra (Leppert et al., 2004; Riedel et al., 2005), ^2h^J_NN_ coupling constants (Brown et al., 2002; Pham et al., 2007; Joyce et al., 2008) and N-H bond lengths (Munowitz et al., 1981; DiVerdi and Opella, 1982; Roberts et al., 1987; Hohwy et al., 2000; Zhao et al., 2001b; Song et al., 2001; White and Hong, 2015; Duong et al., 2019). The latter lengthen exponentially as a function of decreasing N···N separation (Steiner, 1995; Herschlag and Pinney, 2018) with variations of up to ca. 0.05 Å in N-H bond length predicted for typical N···N separations that may be encountered within different DNA bp hydrogen bonding environments (Dingley et al., 1999). Given the relative simplicity and high precision of solid-state NMR ^15^N-^1^H bond length measurements in biomolecules enabled by improved pulse schemes (Hong et al., 1997; Hohwy et al., 2000; Zhao et al., 2001b) and further facilitated by significant, ∼one to two orders of magnitude or larger, enhancements in spectral sensitivity that are afforded by the use of low-temperature DNP technology (Barnes et al., 2008; Jaudzems et al., 2019) we focus here on the application of this approach to DNA systems. Specifically, using a 12-mer DNA duplex containing two central ^13^C,^15^N-enriched WC A-T bps that both adopt HG conformations upon binding of the antibiotic echinomycin (Xu et al., 2018; Conroy et al., 2022) as a model system, we systematically compare details of N-H···N hydrogen bonds for the different A-T base pair conformers within the same DNA sequence context and explore the potential of DNP solid-state NMR to be more broadly applicable toward comparative analysis of hydrogen bonding in different base pairing environments within a range of large DNA complexes that are difficult or impossible to probe by solution-state NMR techniques.

## Results

**FIGURE 1:**
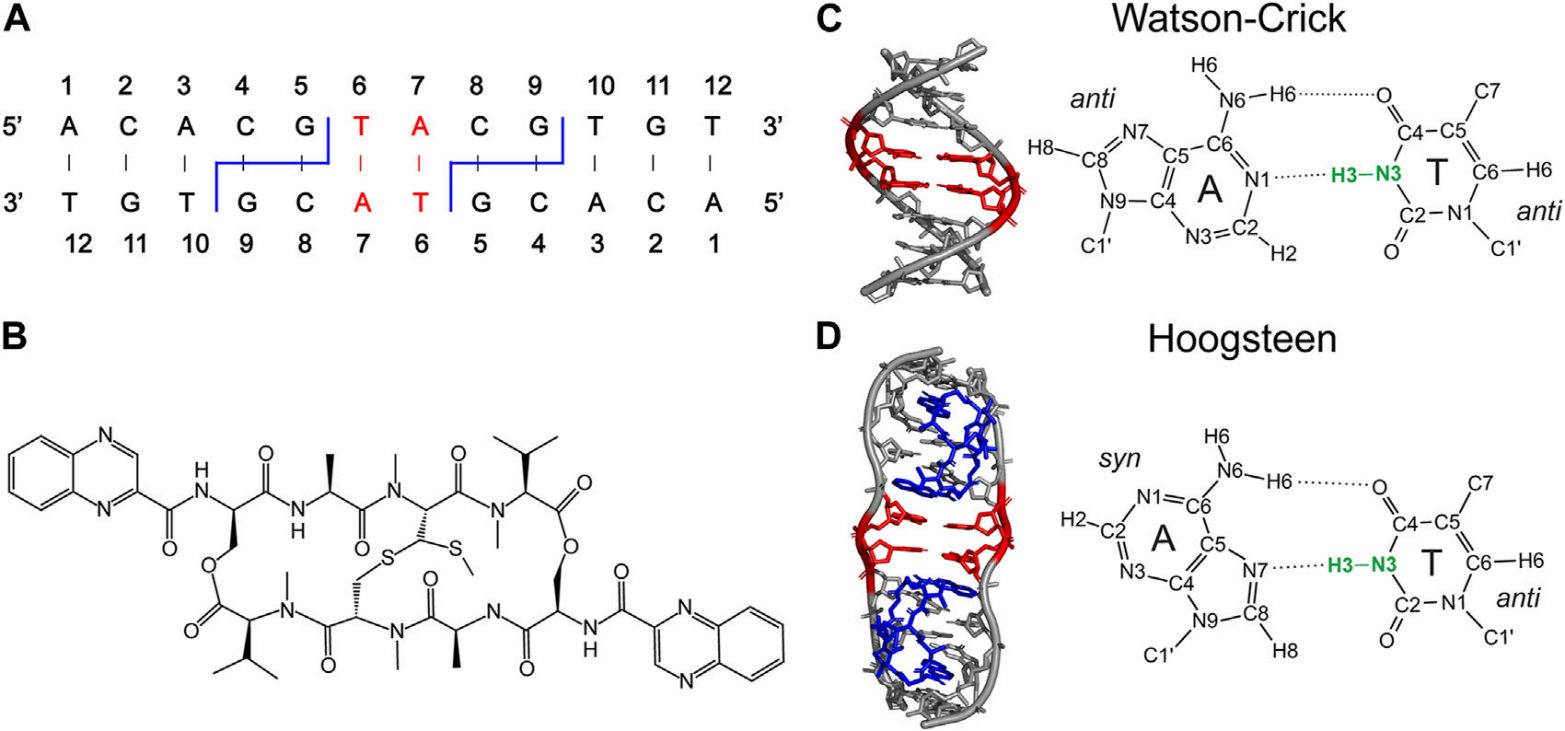
**(A)** Secondary structure of 12-mer DNA, containing ^13^C,^15^N-labeled 6T and 7A residues (red) and 4C-5G and 8C-9G echinomycin binding sites (blue). The binding of echinomycin **(B)** to the 12-mer DNA duplex results in formation of two central A-T Hoogsteen base pairs (red). **(C, D)** DNA duplex structures with the two central A-T base pairs in Watson-Crick **(C)** and Hoogsteen **(D)** conformation highlighted in red and bound echinomycin molecules in **(D)** highlighted in blue. The idealized B-DNA structure with Watson-Crick base pairing in **(C)** was generated using 3DNA (Wijmenga and van Buuren, 1998) and the DNA structure with central A-T Hoogsteen base pairs in **(D)** corresponds to the experimental X-ray crystal structure of the (ACGTACGT)_2_ echinomycin complex (PDB entry 1XVN) (Cuesta-Seijo and Sheldrick, 2005). Thymine N3-H3 bonds, which are the main focus of the present study, are highlighted in bold green.

Quantitative measurements of thymine N3-H3 bond lengths were performed for a ^13^C,^15^N-6T-7A-labeled 12-mer DNA duplex with sequence 5′-ACACGTACGTGT-3′, free and in complex with echinomycin, which features two WC and HG A-T bps in the central TpA step, respectively (Figure 1) (Gilbert et al., 1989; Macaya et al., 1991; Xu et al., 2018; Conroy et al., 2022). For brevity, these samples are referred to here as WC-DNA and HG-DNA. In addition, ^13^C,^15^N-labeled N-acetyl valine (NAV), which contains a single peptide bond, was used to determine the amide N-H bond length under comparable experimental conditions including sample temperature and MAS rate providing an important reference in the context of the present study as described in detail in the Discussion section below.

The N-H bond lengths were probed by using the symmetry-based R18_1_^7^ pulse sequence (Supplementary Figure S1), which enables efficient γ-encoded recoupling of ^15^N-^1^H dipolar interactions with simultaneous ^1^H–^1^H decoupling under MAS (Zhao et al., 2001a; Levitt, 2008) and has successfully been used previously to determine ^13^C/^15^N-^1^H distances as well as relative orientations of dipolar and chemical shift anisotropy (CSA) tensors (Zhao et al., 2001a; Hou et al., 2010; 2011; Mukhopadhyay et al., 2018). Given the well-known sensitivity of the dipolar scaling factor for R-symmetry and other heteronuclear dipolar recoupling sequences to the precise radiofrequency (rf) field amplitude (Hohwy et al., 2000; Zhao et al., 2001b; Pandey et al., 2015; Liang et al., 2021), as illustrated for NAV in Supplementary Figure S2, the R18_1_^7^ ^1^H rf field amplitudes for the WC-DNA, HG-DNA and NAV samples were set to be effectively identical by matching the complete ^1^H rf nutation profiles (Supplementary Figure S3) rather than calibrating a single ^1^H 180° pulse length for each sample. The use of this approach is critical for minimizing potential systematic errors/artefacts in N-H bond lengths extracted for different samples arising from sample-to-sample variations in the R18_1_^7^ ^1^H rf field amplitude (and hence the dipolar scaling factor) as opposed to genuine structural differences between the samples.

**FIGURE 2:**
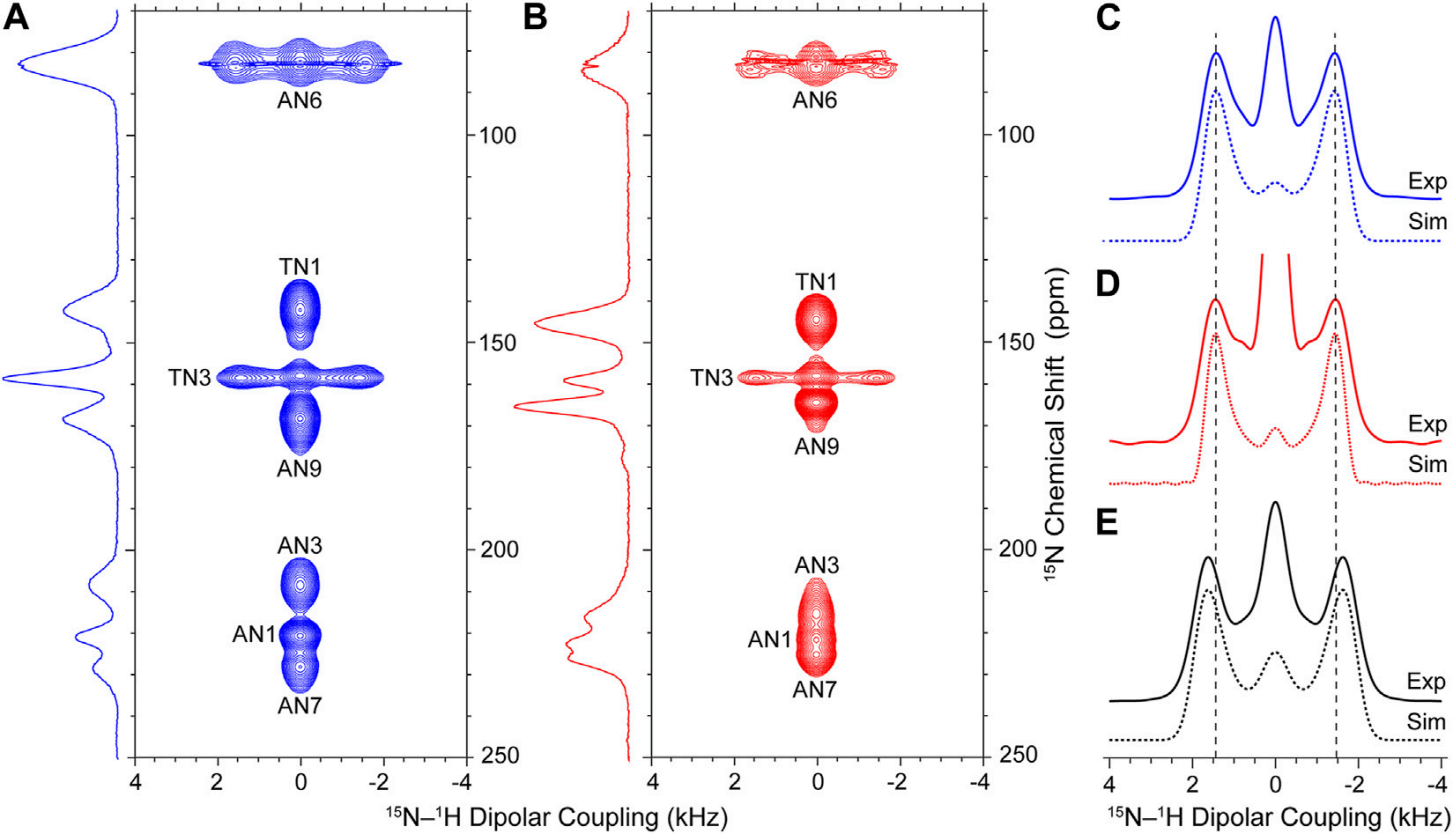
Experimental ^15^N-^1^H dipolar coupling/^15^N chemical shift DNP solid-state NMR correlation spectra for WC-DNA **(A)** and HG-DNA **(B)** recorded with a total experiment time of ∼120 h per spectrum. Reference ^15^N CP-MAS DNP solid-state NMR spectra for WC-DNA and HG-DNA recorded with 2,048 scans each are shown to the left of each panel. **(C–E)** Experimental ^15^N-^1^H dipolar lineshapes (solid lines) and best-fit simulations (dotted lines) for TN3 sites in WC-DNA **(C)** and HG-DNA **(D)** samples (see Supplementary Figure S4 for experimental ^15^N-^1^H dipolar lineshapes for all ^15^N sites) and the amide site in NAV used as a reference **(E)**. The vertical dashed lines in **(C–E)** serve as visual guides. The best-fit simulations were generated within the SIMPSON software package to yield ^15^N-^1^H dipolar couplings/bond lengths as follows: WC-DNA: 10,360 ± 313 Hz/1.055 ± 0.011 Å; HG-DNA: 10,232 ± 323 Hz/1.060 ± 0.011 Å; NAV: 11,659 ± 325 Hz/1.015 ± 0.010 Å. Adjusting the value of the R18_1_^7^ dipolar scaling factor to give an amide peptide N-H bond length of 1.041 Å for NAV, which corresponds to the value predicted by quantum chemistry calculations that take into account zero-point vibrations and librations (Case, 1999), yields TN3-H3 bond lengths of 1.083 Å and 1.087 Å for the WC-DNA and HG-DNA samples, respectively (see text for details).

With the above considerations, we proceeded to record site-resolved ^15^N-^1^H dipolar trajectories for the WC-DNA, HG-DNA and NAV samples by collecting series of ^15^N DNP solid-state NMR spectra using the pulse scheme in Supplementary Figure S1 as a function of increasing R18_1_^7^ time, τ_DIP_. In Figure 2 and Supplementary Figure S4, we show the 2D ^15^N-^1^H dipolar coupling/^15^N chemical shift correlation spectra for the WC-DNA and HG-DNA samples, respectively, which correlate the isotropic chemical shift for each ^15^N site with the frequency domain ^15^N-^1^H dipolar lineshape corresponding to the appropriate time domain dipolar trajectory. These spectra clearly show Pake doublet-like ^15^N-^1^H dipolar lineshapes for the two ^15^N sites (TN3 and AN6) with directly bonded ^1^H atoms, while the remaining ^15^N sites without directly bonded ^1^H exhibit singlet-like lineshapes. For the TN3 site with a single directly bonded ^1^H the splitting between the doublet singularities is directly proportional to the magnitude of the recoupled ^15^N-^1^H dipolar interaction, while for AN6 the dipolar spectrum depends on the magnitudes of dipolar couplings between ^15^N and the two directly bonded ^1^H’s as well as the ^1^H-^15^N-^1^H bond angle which determines the relative orientation of the ^15^N-^1^H dipolar tensors (Hohwy et al., 2000).

To compare the TN3-H3 bond lengths for Watson-Crick and Hoogsteen A-T base pairs in the model 12-mer DNA duplex, we focus our attention on the ^15^N-^1^H dipolar lineshapes for TN3 in the WC-DNA and HG-DNA spectra (Figures 2C, D). A cursory inspection of these lineshapes reveals nearly identical splittings between the doublet singularities indicative of no major variations in the TN3-H3 bond length as a function of A-T base pair conformation for the model DNA duplex. Note that the increase in the intensity of the central “zero-frequency” feature, which is well isolated from the two singularities and stems from secondary effects including rf inhomogeneity, spin relaxation, dipolar couplings to distant ^1^H atoms and ^1^H CSA that is also recoupled by the R18_1_^7^ sequence along with the ^15^N-^1^H dipolar couplings (Supplementary Figure S5) (Hohwy et al., 2000; Zhao et al., 2001b; Hou et al., 2011; Pandey et al., 2015), for TN3 in HG-DNA relative to WC-DNA results from increased partial spectral overlap with the AN9 singlet in the HG-DNA sample. To quantify the TN3-H3 bond lengths for the WC-DNA and HG-DNA samples we fit the experimental ^15^N-^1^H dipolar lineshapes using simulated lineshapes generated for the R18_1_^7^ recoupling sequence within the SIMPSON numerical spin dynamics simulation program (Bak et al., 2000; Tošner et al., 2014). The best-fit simulated ^15^N-^1^H dipolar lineshapes shown in Figures 2C, D correspond to TN3-H3 bond lengths of 1.055 ± 0.011 Å and 1.060 ± 0.011 Å for Watson-Crick vs Hoogsteen A-T bps, respectively, indicating that in the 12-mer DNA duplex samples investigated in the present study the TN3-H3 bond lengths are effectively identical within experimental error irrespective of the base pair conformation. Note that although for each 12-mer DNA duplex sample these TN3-H3 bond lengths correspond to an average value for two isotopically labeled A-T bps, the individual TN3-H3 bond lengths are expected to be effectively identical given the symmetric nature of the DNA duplex in the present study. For comparison, the ^15^N-^1^H dipolar lineshape for NAV shown in Figure 2E clearly displays a larger splitting between the doublet singularities relative to the DNA duplex samples. The best fit of this lineshape yields a peptide amide N-H bond length of 1.015 ± 0.010 Å, which is considerably shorter than the T3 imino N-H bond lengths within Watson-Crick and Hoogsteen A-T DNA base pairs.

## Discussion

The amide N-H bond length of 1.015 ± 0.010 Å obtained in the present study for NAV is consistent within experimental error with the 1.020–1.024 Å N-H distances observed for small peptides using neutron diffraction (Kvick et al., 1977) and the effective N-H bond length of 1.015 ± 0.006 Å determined for the model protein GB3 using NMR measurements of residual dipolar couplings (Yao et al., 2008). Relative to this peptide N-H bond length for NAV, the T3 N-H bond lengths for A-T bps in the 12-mer DNA duplex samples were found to be distinctly longer, by ∼4–5%. Specifically, as noted above, the TN3-H3 bond lengths for the WC-DNA and HG-DNA samples were found to be 1.055 ± 0.011 Å and 1.060 ± 0.011 Å, respectively (c.f., Figure 1), and these values agree reasonably well with the 1.044 Å TN3-H3 distance observed for a Hoogsteen A-T base pair in the neutron diffraction structure of 9-methyladenine/1-methylthymine (Frey et al., 1973) as well as with the ∼1.04–1.08 Å TN3-H3 distances predicted by DFT for Watson-Crick and Hoogsteen A-T base pairs with AN1/AN7···TN3 inter-residue separations ranging from 3.0 to 2.6 Å (Barfield et al., 2001).

It is noteworthy, however, that the peptide N-H bond length of 1.015 Å obtained for the NAV reference sample in the present study is shorter than the effective peptide N-H bond length of 1.041 Å predicted by detailed quantum chemistry calculations that take into account bond vibrations and librations, which due to zero-point motion are expected to be important even at low temperature (Case, 1999), as well as the experimental vibrationally averaged N-H bond lengths of 1.041 ± 0.006 Å determined by NMR measurements of residual dipolar couplings in ubiquitin (Ottiger and Bax, 1998) and ∼1.04–1.06 ± 0.065 Å determined for several crystalline peptides, including NAV, by using quantitative separated local field solid-state NMR experiments performed with careful calibration of the multiple-pulse sequence dipolar scaling factor (Roberts et al., 1987). This discrepancy does not affect the conclusions of the current comparative study, which relates the N-H bond lengths for the different DNA bp conformers to each other and to a well-defined reference peptide N-H bond length determined under comparable experimental conditions including sample temperature, MAS rate and R18_1_^7^ ^1^H rf field. Nevertheless, to assess whether the above noted differences in peptide N-H bond length for NAV result primarily from dependence of the R18_1_^7^ dipolar scaling factor on the exact ^1^H rf field amplitude (Supplementary Figure S2) or from the fact that the NMR measurements were performed at a temperature of ∼100 K rather than ambient temperature we compare in Supplementary Figure S6 the ^15^N-^1^H dipolar lineshapes for NAV recorded at room temperature and at 112 K. While these data reveal a slight (∼1%) increase in the effective N-H bond length at room temperature relative to 112 K, they do not account for the entire difference and are generally consistent with previous low temperature studies of a crystalline peptide which did not reveal significant changes in Cα-Hα bond lengths in the 165–290 K range (Bajaj et al., 2009). This leads to the overall conclusion that, at least in part, the observed discrepancy in N-H bond length for NAV is related to the experimental R18_1_^7^ dipolar scaling factor exceeding the theoretical dipolar scaling factor under the conditions of our study.

To facilitate more direct comparisons of the N-H bond lengths extracted from experimental ^15^N-^1^H dipolar coupling/^15^N chemical shift DNP solid-state NMR correlation spectra to the effective vibrationally averaged values found in the above noted NMR and computational studies, the magnitude of the R18_1_^7^ dipolar scaling factor can be straightforwardly adjusted in the course of data analysis—by multiplying all dipolar couplings extracted from experimental dipolar/chemical shift correlation spectra (c.f., Figure 2) by a factor of 0.926 corresponding to the ratio of dipolar couplings for ^15^N-^1^H distances of 1.041 Å (10,797 Hz) and 1.015 Å (11,659 Hz)—to yield effective N-H bond lengths of 1.041 Å for NAV, 1.083 Å for WC-DNA and 1.087 Å for HG-DNA. Remarkably, these adjusted TN3-H3 bond lengths for WC and HG A-T bps in the model 12-mer DNA duplex are still considerably shorter than the 1.13 Å value obtained for WC A-T (and G-C) bps in B-DNA using early separated local field solid-state NMR experiments (DiVerdi and Opella, 1982), which most likely were also affected by the aforementioned issues related to strong sensitivity of dipolar scaling factor for multiple-pulse homonuclear decoupling/heteronuclear recoupling sequences to the exact ^1^H rf field and/or other experimental parameters.

Irrespective of the exact N-H bond length magnitudes, the key finding of our comparative analysis is that the TN3-H3 bond lengths in WC and HG A-T bps are effectively identical within the same DNA sequence context for the model 12-mer DNA duplex. Given the expected exponential lengthening of the N-H bond in N-H···N hydrogen bonds as a function of decreasing N···N separation (Steiner, 1995; Barfield et al., 2001; Herschlag and Pinney, 2018), under the assumption of approximately linear N-H···N hydrogen bonds this result indicates that N···H hydrogen bond lengths in the WC-DNA and HG-DNA samples are effectively identical. The latter notion is also supported by the fact that TN3-AN7 distances of 2.85 Å observed for the central HG A-T bps in the high-resolution crystal structure of the (ACGTACGT)_2_ echinomycin complex (Cuesta-Seijo and Sheldrick, 2005) match the typical TN3-AN1 distances of 2.81 ± 0.05 Å for WC bps in high-resolution B-DNA crystal structures (Dingley et al., 1999). Finally, we note that in addition to the comparative study of TN3-H3 bond lengths in WC and HG bps for the model 12-mer DNA duplex investigated herein, which yields insights about the relative lengths of the associated AN1/AN7···TH3 hydrogen bonds, the ability of DNP solid-state NMR based experiments to probe N-H bond lengths in different DNA environments with precision of ∼0.01 Å underscores the general utility of this methodology for detailed comparative analysis of hydrogen bonding in a broad range of nucleic acid complexes.

## Materials and methods

### DNA duplex and NAV samples for solid-state NMR

Preparation of WC-DNA and HG-DNA samples for DNP solid-state NMR analysis was described in detail previously (Conroy et al., 2022). Briefly, the 12-mer DNA sequence 5′-ACACGTACGTGT-3′ was synthesized with uniform ^13^C,^15^N enrichment in positions T6 and A7 (c.f., Figure 1). This sequence contains two binding sites for the antibiotic echinomycin at positions C4-G5 and C8-G9. In the absence of echinomycin the sequence forms a canonical Watson-Crick DNA duplex, while stoichiometric binding of echinomycin in a 2:1 molar ratio results in the formation of two HG bps involving residues T6 and A7. The ^13^C,^15^N-labeled DNA duplexes, free and in stoichiometric complex with echinomycin, were prepared as solutions in d_8_,^12^C-glycerol and H_2_O in a 60:40 v/v ratio containing 12 mM AMUPol polarizing agent (Sauvée et al., 2013), 15 mM sodium phosphate, 150 mM NaCl and 0.1 mM EDTA at pH 6.8 with DNA duplex concentrations of 4.1 mM (WC-DNA) and 2.7 mM (HG-DNA). For DNP solid-state NMR measurements, the solutions with volumes of 23.5 μL containing ∼60–100 nanomoles of DNA duplex per sample were transferred to 3.2 mm Bruker sapphire rotors and each rotor was sealed with a silicone plug and ceramic cap. The reference microcrystalline ^13^C,^15^N-labeled NAV sample was described previously (Helmus et al., 2008) and packed in a 3.2 mm Bruker zirconia rotor.

### Solid-state NMR spectroscopy and numerical simulations

NMR measurements were performed on a 600 MHz/395 GHz wide-bore Bruker Avance-III DNP solid-state NMR spectrometer equipped with a gyrotron and a 3.2 mm triple-resonance (HXY) low-temperature MAS probe. The MAS frequency and sample temperature were controlled at 9,921 ± 3 Hz and 112 ± 1 K, respectively, and 130 mA continuous microwave irradiation was applied during the experiments. Recycle delays of 4.6 s were used for the WC-DNA and HG-DNA samples, corresponding to 1.256 times the DNP build-up times determined from saturation-recovery experiments (Conroy et al., 2022), and a 20 s recycle delay was used to record the spectra for NAV.

Measurements of ^15^N-^1^H dipolar couplings were carried out using the pulse scheme shown in Supplementary Figure S1, with parameters similar to those described in an earlier study (Mukhopadhyay et al., 2018). The R18_1_^7^ sequence was incremented on the ^1^H channel during τ_DIP_ up to a maximum evolution time of ∼2 ms, in intervals of 11.2 μs corresponding to two 180° pulses with ∼89.3 kHz rf amplitude and −70°, +70° phase alternation; one full cycle of R18_1_^7^ corresponds to 18 180° pulses applied with −70°, +70° phase alternation within one rotor period (Zhao et al., 2001a). The ^15^N-^1^H dipolar coupling/^15^N chemical shift correlation NMR spectra were recorded in multiple blocks with ∼7 h experiment time each and subsequently combined using nmrglue (Helmus and Jaroniec, 2013), with each block consisting of 180 points in the ^15^N-^1^H dipolar coupling dimension with R18_1_^7^ sequence incremented as described above and 32 scans per point. Spectra were processed in NMRPipe (Delaglio et al., 1995), and experimental time-domain ^15^N-^1^H dipolar trajectories for individual ^15^N sites were extracted using nmrglue (Helmus and Jaroniec, 2013).

The R18_1_^7^ ^15^N-^1^H dipolar trajectories were Fourier transformed in MATLAB to generate the corresponding frequency-domain ^15^N-^1^H dipolar lineshapes, where for ^15^N sites with a single directly bonded ^1^H the splitting between the doublet singularities is directly proportional to the ^15^N-^1^H dipolar coupling magnitude. These ^15^N-^1^H dipolar lineshapes were fit using the Tcl-based optimization package integrated into the SIMPSON numerical spin dynamics simulation software (Bak et al., 2000; Tošner et al., 2014) to extract the ^15^N-^1^H dipolar coupling magnitudes with associated uncertainties, which were subsequently converted to N-H bond lengths and corresponding uncertainties. In addition to the ^15^N-^1^H dipolar coupling and exponential relaxation, the magnitudes of which were optimized during the fitting procedure, the SIMPSON simulations also included ^1^H CSA with the following parameters for TH3 in WC-DNA and HG-DNA (*δ* = 15.4 ppm, *η* = 0.81) (Czernek, 2001) and H^N^ in NAV (*δ* = 8.9 ppm, *η* = 0.75) (Hou et al., 2010).

## Data Availability

The original contributions presented in the study are included in the article/Supplementary Material, further inquiries can be directed to the corresponding author.
