# Supplementary material for: Hydrogen bonding in duplex DNA probed by DNP enhanced solid-state NMR N-H bond length measurements
Source: Front Mol Biosci. 2023 Dec 4;10:1286172. doi: 10.3389/fmolb.2023.1286172 (PMC10726973; doi:10.3389/fmolb.2023.1286172)
Supplement: Supplementary file 1 [file DataSheet1.PDF]

frontiers

Supplementary Material

Hydrogen bonding in duplex DNA probed by DNP enhanced solid-state NMR N-H bond length measurements

Lakshmi Bhai[1], Justin K. Thomas[1], Daniel W. Conroy[1], Yu Xu[2], Hashim M. Al-Hashimi[3], Christopher P. Jaroniec[1]*

[1]Department of Chemistry and Biochemistry, The Ohio State University, Columbus, OH, USA
[2]Department of Chemistry, Duke University, Durham, NC, USA
[3]Department of Biochemistry and Molecular Biophysics, Columbia University, New York, NY, USA

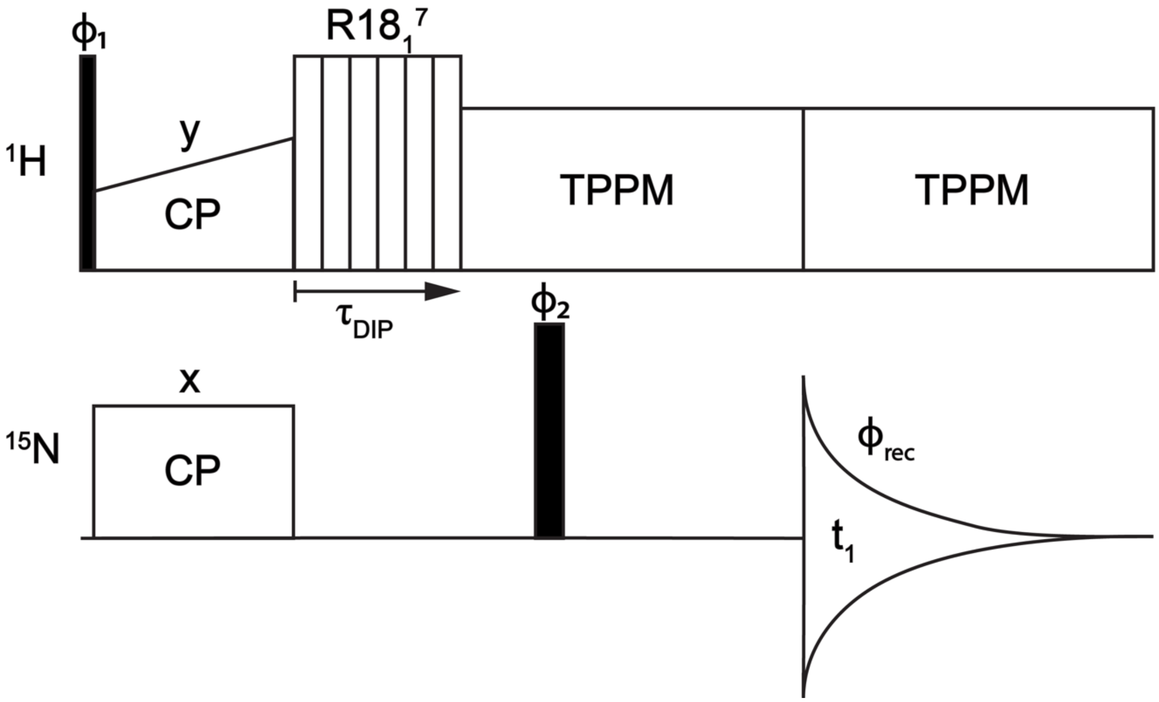

Supplementary Figure S1. Pulse scheme used to record ^{15}N-^{1}H dipolar coupling/^{15}N chemical shift solid-state NMR correlation spectra (Zhao, X. *et al.* (2001) *Chem. Phys. Lett.* 342, 353-361). Narrow and wide black rectangles correspond to $\pi/2$ and π pulses, respectively. The ^{15}N magnetization generated by cross-polarization (CP) from ^{1}H evolves under ^{15}N-^{1}H dipolar interactions recoupled during τ_{DIP} by the $R18_1^7$ pulse sequence as described in the text, with each complete $R18_1^7$ cycle consisting of 18 ^{1}H π pulses applied with -70°, +70° phase alternation within one rotor period. Phase cycling: $\phi_1 = 2(x),2(-x)$; $\phi_2 = x,y$; $\phi_{rec} = x,-x,-x,x$.

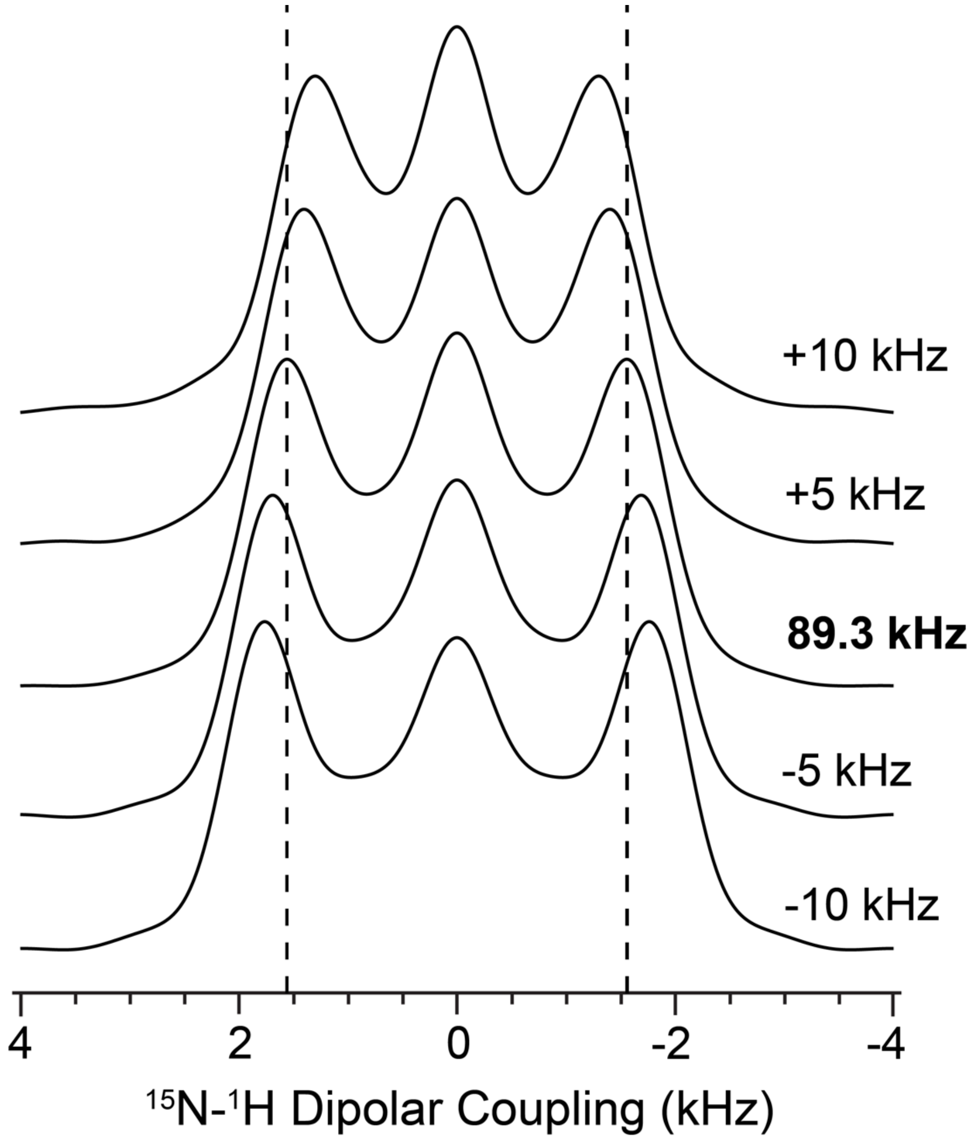

Supplementary Figure S2. Experimental ^{15}N-^{1}H dipolar lineshapes for the amide ^{15}N site in NAV recorded at room temperature as a function of the $R18_1^7$ ^{1}H rf field amplitude ranging from ~79 to ~99 kHz, with the optimal rf field nominally corresponding to 89.3 kHz for the 9,921 Hz MAS rate used in our studies. These measurements show a strong dependence of the dipolar scaling factor for the $R18_1^7$ sequence on the ^{1}H rf field amplitude as noted previously for other R-symmetry sequences (Zhao, X. *et al.* (2001) *J. Am. Chem. Soc.* 123, 11097-11098), which must be taken into account as part of the $R18_1^7$ ^{1}H rf field amplitude calibration procedure (c.f., Supplementary Figure S3) in order to make meaningful comparisons between N-H bond lengths measured for different samples.

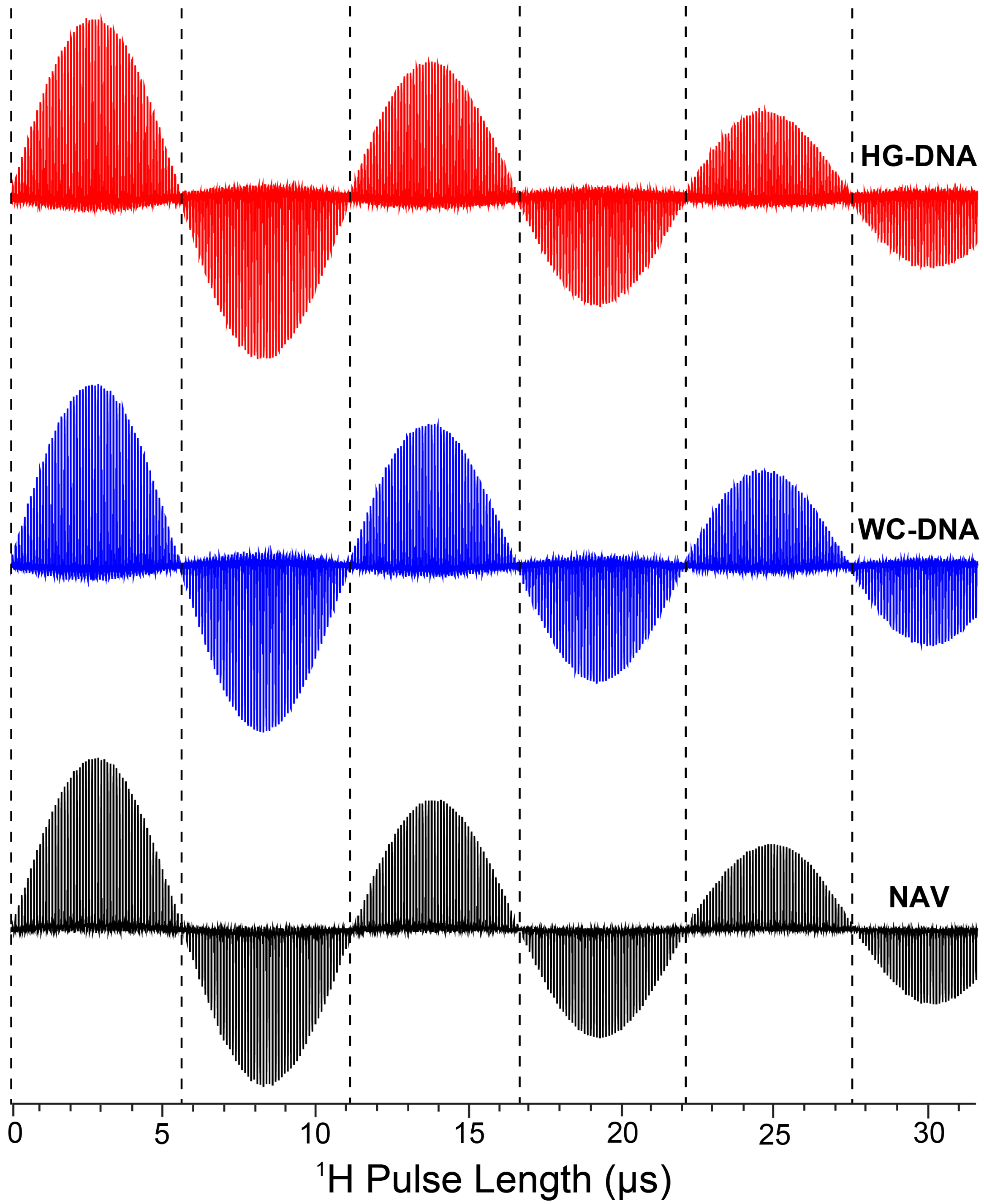

Supplementary Figure S3. Representative ^{1}H nutation profiles recorded at 112 K and used to determine the $R18_1^7$ rf field amplitude for the WC-DNA, HG-DNA and NAV samples. As discussed in the text, this approach ensures that effectively identical $R18_1^7$ rf fields are used for all samples studied and minimizes any potential sample-to-sample differences in measured N-H bond lengths that could arise due to variations in the $R18_1^7$ dipolar scaling factor that can be expected if different $R18_1^7$ rf fields were used for different samples (c.f., Supplementary Figure S2) rather than to genuine structural differences.

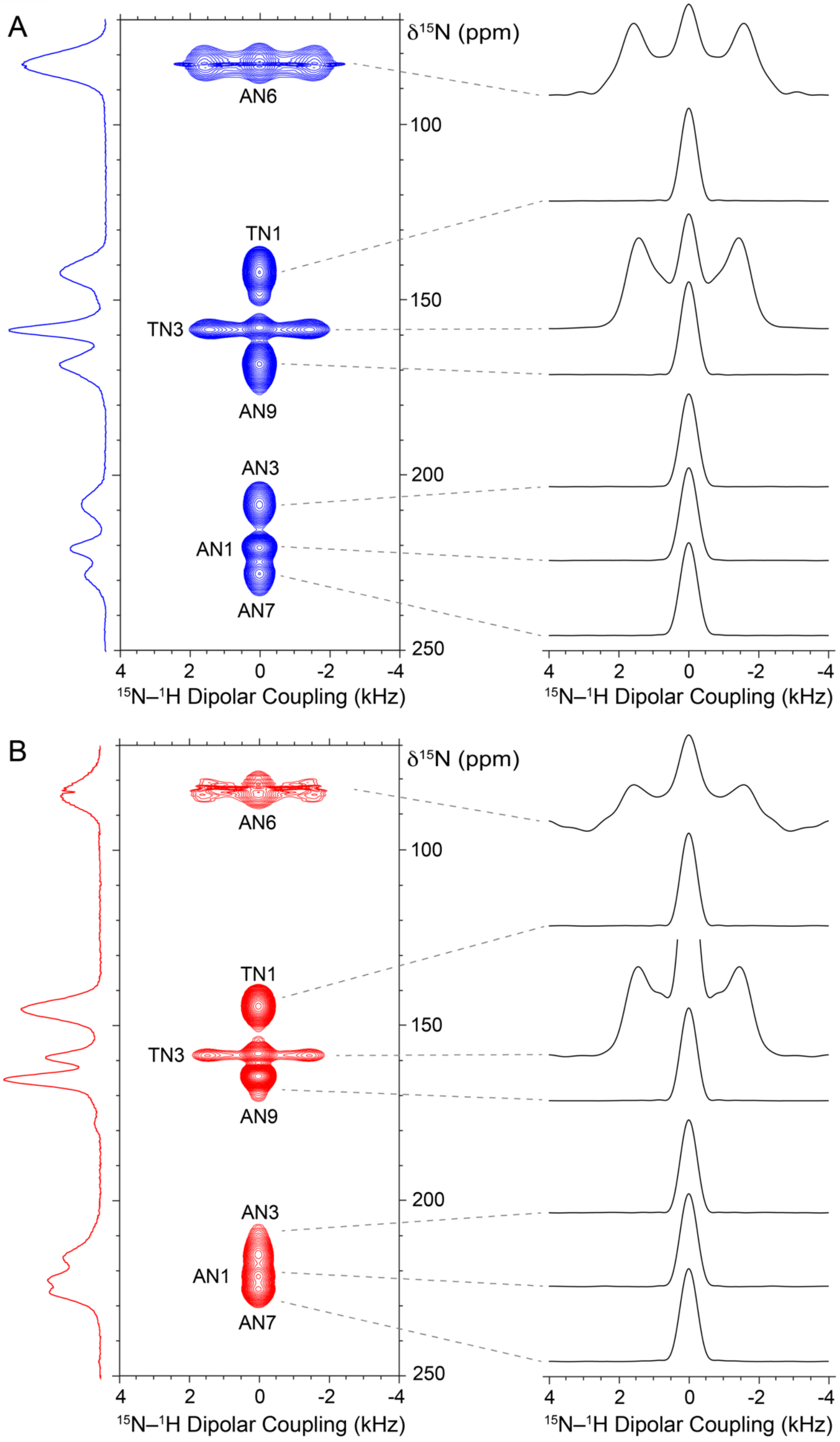

Supplementary Figure S4. Experimental ^{15}N-^{1}H dipolar coupling/^{15}N chemical shift DNP solid-state NMR correlation spectra for WC-DNA (A) and HG-DNA (B) (c.f., Figure 2), showing the ^{15}N-^{1}H dipolar lineshapes for all ^{15}N sites.

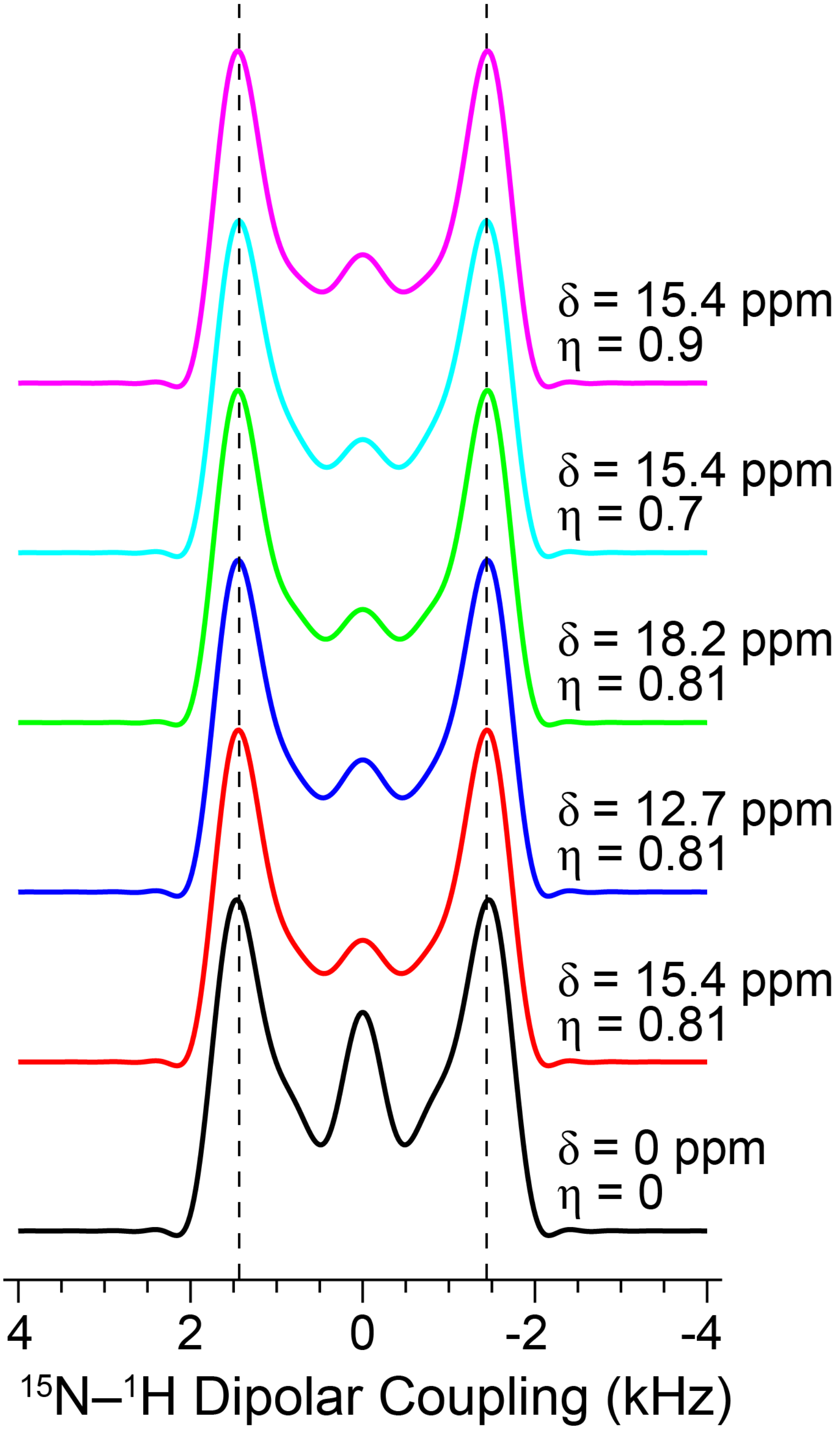

Supplementary Figure S5. Simulated $R18_1^7$ dipolar lineshapes generated using SIMPSON for a ^{15}N-^{1}H dipolar coupling of 10,360 Hz and different ^{1}H CSA parameters, corresponding to typical values expected for thymine H3 in DNA A-T base pairs (Czernek, J. (2001) *J. Phys. Chem. A* 105, 1357-1365).

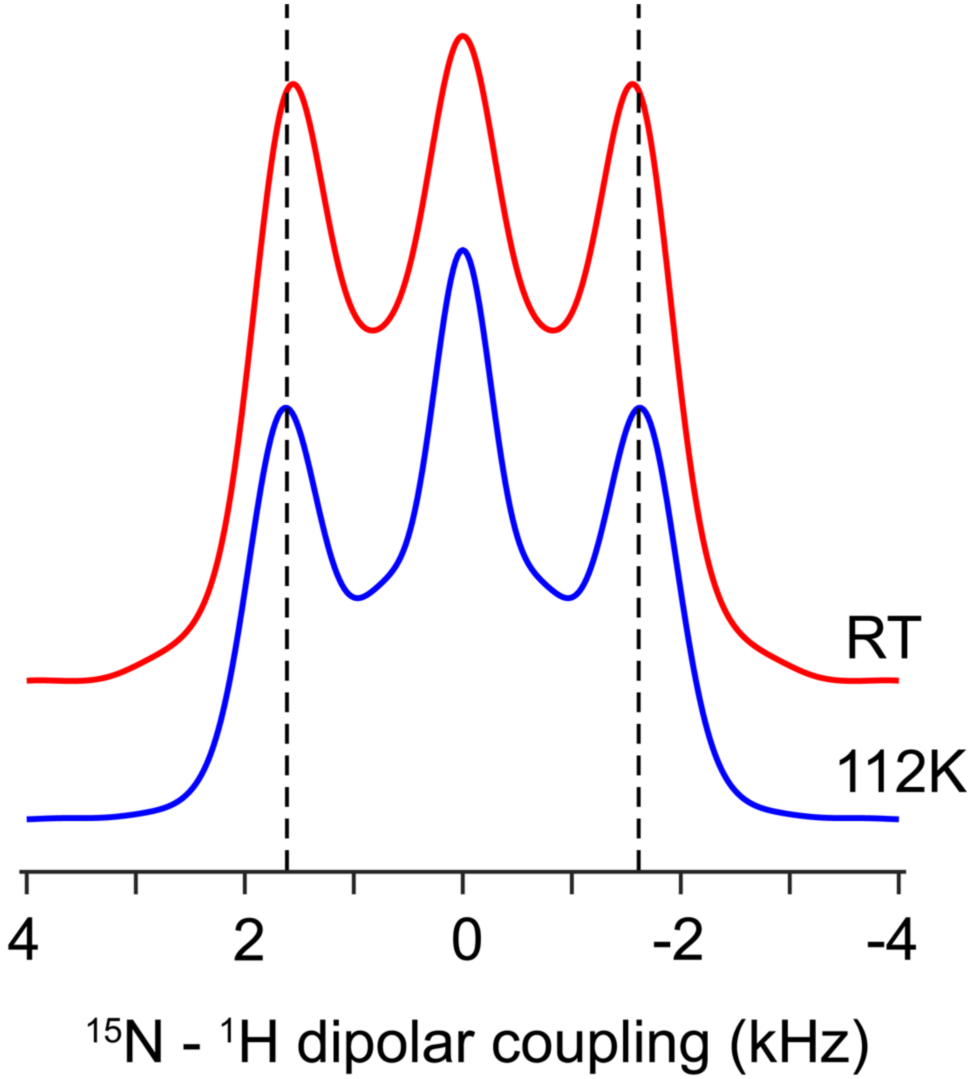

Supplementary Figure S6. Experimental ^{15}N-^{1}H dipolar lineshapes for the amide ^{15}N site in NAV recorded at room temperature (red) and at 112 K (blue) under otherwise identical experimental conditions including, most importantly, the $R18_1^7$ ^{1}H rf field amplitude. These measurements show a slight reduction in the ^{15}N-^{1}H dipolar coupling at room temperature relative to 112 K, consistent with a ~1% increase in the effective N-H bond length from 1.015 Å at 112 K to 1.027 Å at room temperature.
